# Unusual Myoid Differentiation in a Canine Benign Mixed Mammary Tumour

**DOI:** 10.1155/2021/6615256

**Published:** 2021-01-23

**Authors:** Barbara Brunetti, Luisa Vera Muscatello, Louis J. DeTolla, Giancarlo Avallone

**Affiliations:** ^1^Department of Veterinary Medical Sciences, University of Bologna, Italy; ^2^University of Maryland, School of Medicine, Baltimore, MD, USA

## Abstract

This report describes an unusual mesenchymal differentiation in a canine benign mixed mammary tumour. A 13-year-old crossbreed female dog was submitted to surgery to remove an inguinal mammary nodule. The tumour was composed of mammary epithelium and mesenchymal populations, not only of cartilage and bone but also of myoid cells. PTAH demonstrated cross striation of striated muscle, and immunohistochemistry highlighted striated muscle expressing desmin and calponin, and smooth muscle expressing desmin, SMA, and calponin. The tumour was diagnosed as a benign mixed tumour with leio- and rhabdomyoid differentiation. There was no tumour recurrence after one year of clinical follow-up. In conclusion, the well-differentiated features of leiomyocytes and rhabdomyocytes and the growth pattern define the benign origin of the mesenchymal component, which has been confirmed by a benign outcome; therefore, the knowledge of this kind of differentiation is helpful to avoid misdiagnoses.

## 1. Introduction

Canine benign mixed tumour and carcinoma-mixed type are neoplasms with more than two cellular populations [1]. The tumours are composed of benign or malignant epithelial neoplastic cells, myoepithelial cells, and foci of cartilage and/or bone and/or adipose tissue with no atypia [1]. The origin of cartilage and bone in these tumours is still debated with some speculating they could arise from metaplastic changes in epithelial cells [2] or from interstitial stromal cells [3] while a number of other authors claim they originate from myoepithelial cells [4–6]. Myoid differentiation is a rare feature of human metaplastic carcinoma of the breast [7], and to our knowledge, it has never been described in a mammary tumour of animals. Cartilaginous and osseous metaplasia is not exclusive to mammary mixed tumour but can also occur in canine cutaneous mixed apocrine adenoma or carcinoma and mixed ceruminous adenoma and carcinoma. In complex (mixed) apocrine adenoma, the mesenchymal component may occasionally have a leiomyomatous appearance characterized by bundles of elongated cells with eosinophilic cytoplasm and no surrounding stroma [8]. Metaplastic carcinoma with mesenchymal differentiation of the human breast is characterized by an admixture of mesenchymal components, including chondroid, osseous, rhabdomyoid, and even neuroglial differentiation, with carcinomatous areas, which can be in the form of glandular tubules, solid clusters, and/or foci of squamous differentiation [7].

The aim of this report is to histologically describe and histochemically and immunohistochemically characterize the unusual feature of myoid differentiation in a benign mixed mammary tumour and its correlation with the biological behaviour.

## 2. Case Presentation

A 13-year-old crossbreed female dog was submitted to surgery to remove multiple nodules: one nodule of 1.5 × 1 cm in the right cranial thoracic mammary gland, a second nodule of 1 cm in the right inguinal gland region, and a third nodule of 10 × 7 cm in the left inguinal gland.

Sections were obtained from all the nodules and stained with haematoxylin and eosin (HE), Masson's trichrome, and phosphotungstic acid haematoxylin (PTAH). Immunohistochemistry (IHC) was performed on serial sections using antibodies to vimentin, cytokeratin (CK) 19, CK 14, p63, desmin, alpha-smooth muscle actin (SMA), and calponin. All specifications are reported in Table 1.

**Figure 1:**
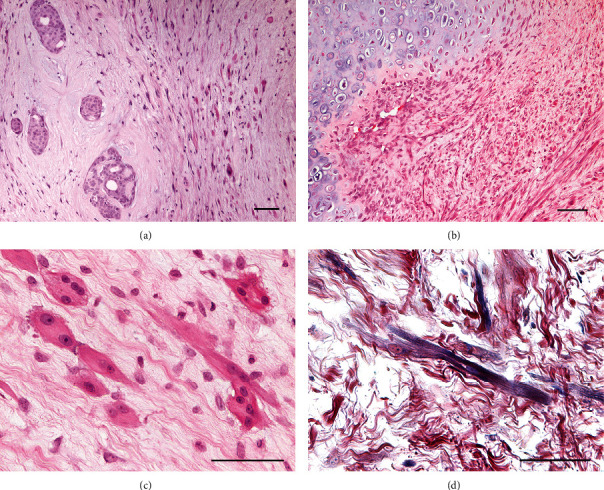
(a) Tubules of epithelial cells without feature of atypia (left) and scattered myoid spindle cells (right), HE, 10x, scale bar 100 micron. (b) Lobules of hyaline cartilage with well-differentiated chondrocytes, surrounded by a moderate cellular population of elongated myoid cells embedded in a moderate amount of myxoid extracellular matrix, HE, 10x, scale bar 100 micron. (c) Giant spindle multinucleate cells, 50-70 micron, with abundant eosinophilic cytoplasm and cross striation (strap-like cells or rhabdomyoblasts), HE, 40x, scale bar 100 micron. (d) Striated muscles are highlighted in blue, with prominent cross striations, PTAH, 40x, scale bar 100 micron.

**Figure 2:**
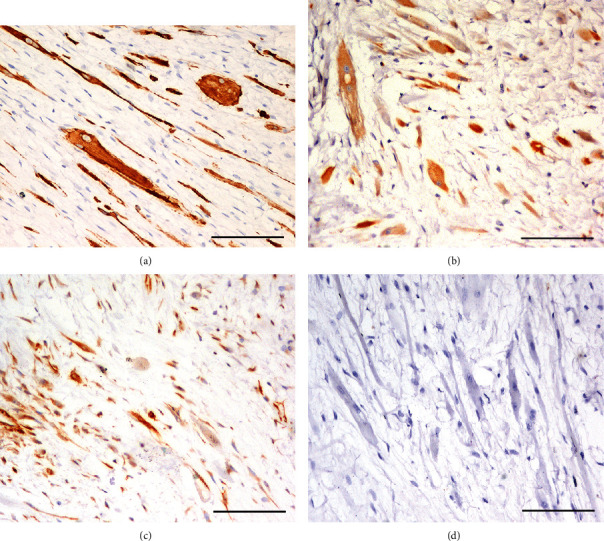
(a) Desmin expression of both thin elongated smooth muscle cells and large multinucleated striated muscle cells. IHC, 20x, scale bar 100 micron. (b) Calponin expression of both thin elongated smooth muscle cells and large multinucleated striated muscle cells, IHC, 20x, scale bar 100 micron. (c) Smooth muscle actin in elongated smooth muscle cells but not in the large striated muscle cells, IHC, 20x, scale bar 100 micron. (d) p63 negative in both smooth muscle and striated muscle cells, IHC, 20x, scale bar 100 micron.

Histologically, the left inguinal mammary mass was well-demarcated, not encapsulated, expansive, and moderately cellular. It was composed of well-differentiated mesenchymal components (cartilage, bone, and smooth and striated muscle) and less than 5% of mammary epithelium. The lesion was multilobular with round nodules composed of poorly organized hyaline cartilage that surrounded bone tissue. Around and between the nodules, there were bundles of mesenchymal cells embedded in a loose myxoid stroma (Figures 1(a) and 1(b)). The mesenchymal cells were 15-25 micron, from spindle to round, with cigar-shaped nuclei. Occasionally, there were 50-70 micron giant spindle to polygonal binucleate or multinucleate cells with abundant eosinophilic cytoplasm with cross striation (strap-like cells or rhabdomyoblasts) (Figure 1(c)). Rare tubules of epithelial cells were admixed to the mesenchymal components. Epithelial cells were 20-25 micron, polygonal with distinct margins, an intermediate nucleus/cytoplasmic ratio, eosinophilic cytoplasm, and a vesicular oval nucleus with a small nucleolus. Anisocytosis and anisokaryosis were mild. Mitoses were rare. PTAH demonstrated cross striation of striated muscle (Figure 1(d)). Masson's trichrome highlighted collagen in blue and muscle fibres in red. IHC showed a strong cytoplasmic expression to vimentin in all the mesenchymal tumour cell populations. These same cells failed to stain for CK19, CK14, and p63. Striated muscle expressed desmin (Figure 2(a)) and calponin (Figure 2(b)), while smooth muscle also expressed SMA (Figure 2(c)). The epithelial component was CK19-positive and associated with a layer of myoepithelium expressing calponin, CK14, and p63 (Figure 2(d)).

Additionally, the neoplasm in the right inguinal mammary gland was a mixed-type carcinoma and the right inguinal mass as a cutaneous lipoma.

All observed mesenchymal components of the tumour (cartilage, bone, and smooth and striated muscle) were well-differentiated and organized in nodules, with no signs of cellular atypia, and therefore were considered of metaplastic origin. Moreover, the morphologic features of epithelial cells were consistent with a benign neoplasm. Based on these findings, we suggest the diagnosis of a canine benign mixed mammary tumour with smooth and striated muscle differentiation.

One year after surgery, the dog was in good health and showed no recurrences.

## 3. Discussion

The presence of neoplastic epithelial cells, even if minimal, ruled out the diagnosis of a purely mesenchymal neoplasm. In the latter case, the most likely diagnosis would have been benign mesenchymoma, based on the identification of three or more differentiated mesenchymal cell phenotypes in the same neoplasm [9]. Benign mesenchymoma of the mammary tissue is an extremely rare tumour described in human medicine [10] and never reported in the canine mammary gland.

Metaplastic carcinoma of the human breast encompasses a group of tumours characterized by differentiation of the neoplastic epithelium into squamous cells and/or mesenchymal-looking elements, including but not restricted to spindle, chondroid, osseous, and rhabdomyoid cells. These neoplasms may be either entirely composed of metaplastic elements or a complex admixture of carcinoma and metaplastic areas [7]. Malignant lesions of the breast are not the only tumours that show myoid differentiation: rarely fibroadenoma of the breast can show a proliferation of exuberant smooth muscle cells of unclear origin. Apart from the erector muscle of the nipple, smooth muscle cells are usually absent in normal mammary stroma. Therefore, smooth muscle cells in fibroadenomas have been interpreted as a metaplastic process originating from stromal fibroblasts, myofibroblasts, and myoepithelial cells [11]. Another tumour with muscle differentiation is myoid hamartoma of the breast, defined as a tumour-forming mass of disorganized but well-differentiated cells autochthonous to their place of origin [12]. Myoid hamartoma is considered a rare variant of mammary hamartoma containing extensive foci of spindle cells along with the natural breast tissue cells and supporting tissues. In general, the smooth muscle cell component in myoid hamartoma is thought to originate from the smooth muscle cells of vessels, the nipple, undifferentiated breast tissue stroma, or myoepithelial cells [12]. In 1980, Eusebi et al. described three cases of benign smooth muscle cell metaplasia in benign breast tumours [13]. They considered the hypothesis of a hamartomatous origin of the smooth muscle cells in the breast stroma, or a proliferation from the nipple or blood vessel walls or the breast stroma's capacity for multipotential differentiation. All these hypotheses can be considered plausible in the canine species in the present benign mixed tumour.

The treatment of choice for mammary tumour is surgical excision, and one year after surgery, the dog is in good health, without any disease relapse.

To the best of our knowledge, this is the first report of leio- and rhabdomyoid differentiation in a canine benign mixed tumour. The features of leiomyocytes and rhabdomyocytes and the growing pattern define the benign origin of the mesenchymal component, which is confirmed by a benign biological behaviour; therefore, the knowledge of this kind of differentiation is helpful to avoid diagnostic pitfalls.

## Figures and Tables

**Table 1:** Antibody details.

Antibody	Clone	Source	Dilution
Vimentin	V9	Dako	1 : 600
Cytokeratin 19	BA 17	Histo-Line Laboratories	1 : 400
Cytokeratin 14	Ab1	Bioptica	1 : 600
p63	4A4	Dako	1 : 200
Desmin	DE-R-11	Santa Cruz	1 : 100
Alpha smooth muscle	1A4	Dako	1 : 450
Calponin	Calp	Dako	1 : 2000
